# A Cross-Sectional Study Assessing the Suitability of ChatGPT and DeepSeek AI for Generating Patient Education Guides on Imaging Modalities in Stroke

**DOI:** 10.7759/cureus.92066

**Published:** 2025-09-11

**Authors:** Mohammed Hussain, Mohammed Mobasshir Hassan, Tania Taj, Viraj Shah

**Affiliations:** 1 Respiratory Medicine, Royal Stoke University Hospital, Stoke-on-Trent, GBR; 2 Medicine, Royal Stoke University Hospital, Stoke-on-Trent, GBR; 3 Internal Medicine, Al Wakra Hospital, Hamad Medical Corporation, Al Wakra, QAT; 4 Radiology, Dr. D.Y. Patil Medical College, Hospital and Research Centre, Pune, IND

**Keywords:** artificial intelligence, chatgpt, deepseek ai, diffusion-weighted imaging, digital subtraction angiography, non-contrast ct

## Abstract

Introduction

Patient education plays a critical role in stroke care and management. It helps patients understand their health, diagnosis, diagnostic modalities, and treatment and improves their overall experience. With the integration of AI tools into healthcare, patient education has become efficient and easily accessible, becoming a powerful asset in healthcare.

Methodology

In this cross-sectional study, two artificial intelligence (AI) tools, namely, ChatGPT (OpenAI, San Francisco, California, United States) and DeepSeek AI (DeepSeek, Hangzhou, Zhejiang, China), were prompted to create patient education guides on three imaging modalities, that is, digital subtraction angiography (DSA), non-contrast computed tomography (CT), and diffusion-weighted imaging (DWI), for stroke cases. Both responses were assessed for variables such as number of words, number of sentences, average words per sentence, ease score, grade level, and average syllables per word using the Flesch-Kincaid calculator. The readability and similarity scores were assessed by the modified DISCERN score and Quillbot, respectively. Statistical analysis was done using R version 4.3.2 (R Foundation for Statistical Computing, Vienna, Austria).

Results

In generating patient education materials for non-contrast CT, DW-MRI, and DSA in stroke care, ChatGPT and DeepSeek AI showed similar performance across grade level, ease score, similarity, and reliability, with no statistically significant differences. ChatGPT often produced slightly higher grade levels, while DeepSeek AI had higher ease scores for some modalities. Similarity percentages varied by topic but averaged equally, and reliability was uniformly high. Linguistic features showed only minor, non-significant differences.

Conclusions

Both ChatGPT and DeepSeek AI performed similarly in generating patient education guides based on ease of understanding and readability. These results suggest that either AI tools can be effectively used for patient education in this context.

## Introduction

Stroke is a leading neurological emergency that occurs due to either an obstruction or rupture of a cerebral blood vessel, resulting in compromised blood flow and subsequent neuronal injury. Globally, stroke is one of the foremost causes of mortality and long-term disability, particularly among older adults and individuals with cardiovascular risk factors [1,2]. The outcome of a stroke is highly time-sensitive, with prompt diagnosis and early intervention being crucial to minimize irreversible brain damage and functional decline. In this context, patient education is vital: it enhances awareness of symptoms, supports timely medical attention, and promotes adherence to secondary prevention strategies, all of which contribute to improved recovery outcomes [3].

With the growing integration of digital tools in healthcare, artificial intelligence (AI) has emerged as a potential adjunct in patient communication and education. Among recent developments, large language models (LLMs) such as ChatGPT (OpenAI, San Francisco, California, United States) and DeepSeek AI (DeepSeek, Hangzhou, Zhejiang, China) have shown promise in generating health information that is both accessible and easy to understand. These tools can simplify complex medical terminology and tailor their responses based on user input, offering a personalized approach to health communication. Importantly, they can provide consistent, repetitive explanations without the limitations of human time or fatigue, making them valuable for reinforcing key concepts [2,3].

ChatGPT, developed by OpenAI, has been examined for its ability to respond accurately to medical questions and assist in creating patient-centered educational material [4-6]. DeepSeek AI, a newer language model optimized for clinical contexts, is specifically designed to interpret and translate radiology findings into friendly and easy-to-understand explanations, with potential utility in bridging communication gaps in diagnostic imaging [7]. Given that stroke diagnosis often relies heavily on imaging modalities, such as non-contrast computed tomography (CT), magnetic resonance imaging (MRI), and CT/MR angiography, patient understanding of these procedures is critical but often inadequate due to the technical nature of radiological language.

As the demand for accurate, timely, and understandable health information increases, particularly in acute conditions like stroke, AI-generated tools may help augment traditional educational approaches. This study aims to evaluate and compare the effectiveness of ChatGPT and DeepSeek AI in generating patient education content specifically related to imaging modalities used in stroke care, focusing on the clarity, accuracy, and accessibility of information.

## Materials and methods

A cross-sectional original research study was conducted over one week, from March 1 to March 8, 2025. Since this study did not involve human participants or identifiable personal data, it was exempt from ethics committee review and approval.

Three commonly used imaging modalities for the diagnosis of stroke cases were selected: digital subtraction angiography (DSA), non-contrast CT, and diffusion-weighted imaging (DWI). Two AI tools, namely, DeepSeek AI and ChatGPT version 4.0, were accessed on March 8, 2025, to generate responses to three prompts as follows: (1) "Write a patient education guide for digital subtraction angiography", (2) "Write a patient education guide for non-contrast CT", and (3) "Write a patient education guide for diffusion-weighted imaging".

All six responses were collected and compiled into a Microsoft Word document (Microsoft Corporation, Redmond, Washington, United States) for further statistical analysis. The texts produced by the tools were assessed for number of words, number of sentences, average words per sentence, ease score, grade level, average syllables per word, and similarity and reliability scores.

The Flesch-Kincaid calculator was used to obtain word and sentence counts, ease of understanding (Flesch-Kincaid grade level), and readability (Flesch Reading Ease score). The Quillbot plagiarism tool was used to calculate the similarity percentage. Reliability of the scientific text was evaluated using the modified DISCERN (mDISCERN) score by the study authors, who are medical professionals with experience in research methodology and scientific writing. The mDISCERN is a 5-point Likert scale adapted from a validated tool for evaluating written health information. In this scale, a score of 0 or 1 is assigned for each criterion, that is, precision/clarity, reliability, balance, source, and uncertainty, with higher scores indicating greater reliability [8].

Graded data were exported to a Microsoft Excel sheet, and statistical analysis was performed using R version 4.3.2 (R Foundation for Statistical Computing, Vienna, Austria). Comparisons between ChatGPT and DeepSeek AI outputs were made using an unpaired t-test, with p-values of <0.05 considered statistically significant.

## Results

**Figure 1:**
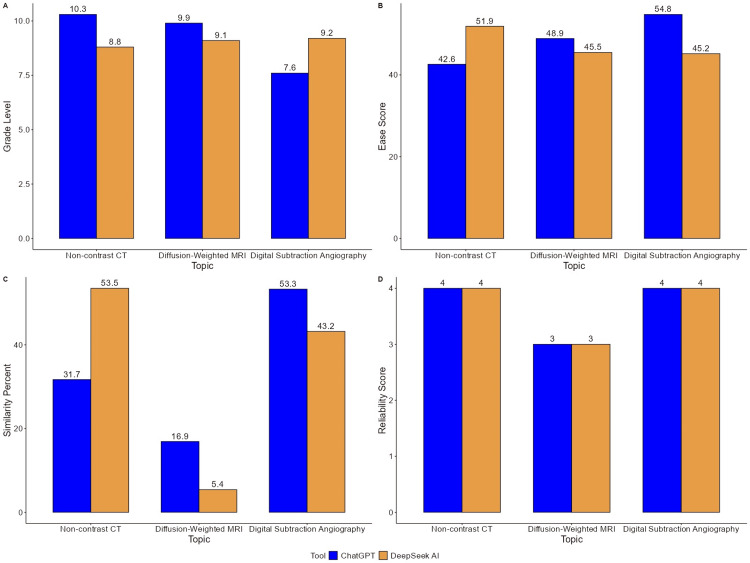
Graphical representation of comparison between grade level, ease score, similarity percent, and reliability score, for the patient education guide generated by ChatGPT and DeepSeek AI

This study assessed and compared the effectiveness of ChatGPT and DeepSeek AI in generating patient education materials for three imaging modalities used in stroke care: non-contrast CT, DW-MRI, and DSA. Evaluation criteria included readability, content similarity, reliability, and various linguistic features, with data derived from both graphical analysis (Figure 1) and tabulated statistics (Table 1).

**Table 1:** Characteristics of responses generated by ChatGPT and DeepSeek AI ^+^t-test. P-values <0.05 are considered statistically significant. AI: artificial intelligence

Variables	AI tools				Statistical analysis
	ChatGPT		DeepSeek AI		P-value^+^
	Mean	Standard deviation	Mean	Standard deviation	
Words	564.00	285.24	611.67	73.12	0.793
Sentences	48.67	14.19	64.00	12.49	0.233
Average words per sentence	11.27	2.94	9.70	1.14	0.437
Average syllables per word	1.73	0.06	1.77	0.06	0.519
Grade level	9.27	1.46	9.03	0.21	0.809
Ease score	48.77	6.10	47.53	3.78	0.781
Similarity %	33.97	18.31	34.03	25.33	0.997
Reliability score	3.67	0.58	3.67	0.58	1.000

Grade level

Analysis of reading complexity indicated that ChatGPT generally produced content at a slightly higher grade level compared to DeepSeek AI. For non-contrast CT, the readability grade level was 10.3 for ChatGPT and 8.8 for DeepSeek AI. A similar trend was observed in the DW-MRI content (9.9 vs. 9.1, respectively). However, in the DSA section, DeepSeek AI yielded a higher grade level (9.2) compared to ChatGPT (7.6), suggesting variability based on topic. These trends are consistent with the overall mean grade levels reported in Table 1, where ChatGPT averaged 9.27 and DeepSeek AI 9.03, with the difference lacking statistical significance (p=0.809).

Readability

Readability, as measured by ease score, varied between the two tools depending on the imaging modality. DeepSeek AI provided more accessible content for non-contrast CT (ease score: 51.9 vs. 42.6 for ChatGPT) and DSA (54.8 vs. 45.2), while ChatGPT had a marginal advantage in the DW-MRI section (48.9 vs. 45.5). Nonetheless, when averaged across topics, the ease scores for ChatGPT (48.77) and DeepSeek AI (47.53) were very similar, with no statistically significant difference noted (p=0.781), as shown in Table 1.

Similarity percentage

Notable differences emerged in content similarity scores, which reflect overlap with pre-existing sources. For non-contrast CT, DeepSeek AI exhibited a higher similarity percentage (53.5%) compared to ChatGPT (31.7%). The pattern reversed for DW-MRI, with ChatGPT scoring higher (16.9%) than DeepSeek AI (5.4%). For DSA, ChatGPT again recorded greater similarity (53.3%) versus DeepSeek AI (43.2%). Despite these topic-level fluctuations, the overall mean similarity scores for both tools were nearly identical, 33.97% for ChatGPT and 34.03% for DeepSeek AI, and statistically indistinct (p=0.997).

Reliability

Both tools demonstrated a high degree of factual accuracy across all modalities. Reliability scores were consistent: 4 out of 4 for both tools in the non-contrast CT and DSA sections and 3 out of 3 for the DW-MRI content. As indicated in Table 1, the mean reliability score for each AI system was 3.67, with no observable difference between them (p=1.000), indicating parity in terms of content correctness and clinical relevance.

Linguistic characteristics

Additional analysis of linguistic features, including word count, sentence length, and lexical complexity, revealed minor variations between the tools. ChatGPT produced slightly shorter responses on average (564 vs. 611.67 words) but used longer sentences (average of 11.27 words per sentence compared to 9.70 for DeepSeek AI). However, none of these differences were statistically meaningful. The average syllables per word were nearly identical (1.73 vs. 1.77), reinforcing the finding that both tools maintained a similar level of language complexity.

Taken together, these findings suggest that while both AI models perform comparably in generating reliable and structured patient education material, their outputs differ subtly depending on the specific imaging topic, particularly in terms of readability and content originality.

## Discussion

This cross-sectional study assessed the suitability of ChatGPT and DeepSeek AI for generating patient education guides on imaging modalities in stroke cases. It revealed that there is no significant difference between the responses generated by both AI tools.

Neuroimaging is crucial in stroke care, as early diagnosis and treatment can prevent serious outcomes. However, individuals with limited health literacy may be less prepared for neuroimaging procedures, which can negatively impact the quality of diagnostic outcomes [9,10]. Patient education is essential in helping patients to understand their diagnosis and treatment, alleviate anxiety and fear, and improve their healthcare experience. Patient education improves their health literacy and enhances their informed decision-making [11].

The American Medical Association (AMA) and the US Department of Health and Human Services (USDHHS) recommend that patient education materials be written at a fifth- to sixth-grade reading level to ensure they are easily understood by the general public [12]. Patient education guides should be written in clear, simple language to ensure they are understandable by individuals with different literacy levels. AI can be used to support this goal by providing patients with accessible information, empowering them to make informed medical decisions. This is especially important for stroke patients, where quality educational materials can significantly support recovery. However, the effectiveness of these guides largely depends on both their accuracy and readability [13,14].

This study aimed to assess ChatGPT and DeepSeek AI in generating patient education guides based on ease of understanding (grade level), readability (ease score), similarity, and reliability. The Flesch Reading Ease score measures how readable a text is and gives an estimate of the education level someone needs to read it easily. The average ease score for ChatGPT was 48.77, and for DeepSeek AI, it was 47.53, both falling within the "difficult" range, which corresponds to college-level reading material, indicating that the content produced may be challenging for the general public to read and requires a higher level of reading skills. This is similar to the study by Behers et al., where the ease score for ChatGPT was 31, depicting a college reading level. ChatGPT demonstrated a slightly higher ease score compared to DeepSeek AI, indicating an easier readability in comparison [15]. 

The Flesch-Kincaid grade level tells you what education level someone should have completed to understand a specific text. The average grade level for ChatGPT was 9.27, and for DeepSeek AI, it was 9.03, respectively, indicating that both AI tools generated content suitable for high school-level understanding. DeepSeek AI had a slightly lower grade level score, suggesting that its content was marginally easier to understand.

However, neither difference was statistically significant as the p-values for both variables were greater than 0.05, suggesting that there is no statistical difference in ease score or grade level between the responses generated by both AI tools. Despite their great potential, AI tools have been found to have problems with plagiarism and have been unable to provide accurate references. This presents doubts regarding the usefulness, accuracy, and integrity of AI in health content writing [16]. Plagiarism not only violates the fundamental principles of originality and credibility but also threatens the foundation of ethical research practices [17]. In this study, QuillBot's plagiarism checker, an online tool that compares text against a large database, showed similarity scores of 33.97% for ChatGPT and 34.03% for DeepSeek AI, with no significant difference. Similarity percentage shows how much of the text matches published content. While both tools produced mostly original content, the results still underline the importance of human review in healthcare materials.

Reliability of the contents generated was analyzed through the mDISCERN score, which is an updated version of the original DISCERN tool, tailored to assess the quality of written health information. It also evaluates online health content based on clarity, relevance, and evidence-based accuracy. It is evaluated on a scale from 1 to 5, with 5 indicating higher reliability [18]. The average DISCERN score for ChatGPT and DeepSeek AI is 3.67, which indicates both tools have produced similar reliable content.

Overall, the findings of this study suggest that both AI tools perform similarly across all variables, including grade level, ease of readability, similarity percentage, and reliability. Saji et al. similarly found no significant differences between ChatGPT and DeepSeek AI in readability, reliability, similarity, or ease of understanding. This suggests that while AI tools are promising, human oversight remains necessary to ensure their content is clear, accurate, and suitable for patients [18].

Limitations

This study has several limitations that should be considered when interpreting the findings. First, only two AI tools, that is, ChatGPT and DeepSeek AI, were evaluated, and results may not be generalizable to other language models. Second, the cross-sectional design provides a snapshot in time; because AI models are continuously updated, outputs may differ if the study is repeated in the future. Third, only a single standardized prompt was used for each imaging modality. While this ensured fairness across tools, it does not capture the full variability of AI responses, which may change with different wording or repeated runs. Fourth, evaluation of reliability using the mDISCERN score introduces subjectivity, as inter-rater reliability was not formally assessed. Similarly, the plagiarism analysis relied on Quillbot, and version-specific settings were not detailed; however, the tool was applied consistently across both models, preserving fairness in comparison. Fifth, raw AI-generated texts were not included as supplementary files, which limits strict reproducibility, although the methodology has been described transparently to allow replication of the process. Finally, while readability was measured using validated indices, both tools produced content at high school-college reading levels, which remain above the AMA and USDHHS recommendations of a fifth to sixth grade level for patient education. No testing was performed with actual patients or lay readers, so practical comprehension and clinical applicability were not assessed. Despite these limitations, the study provides valuable preliminary insights into the methodological comparability of two AI tools in generating patient education materials.

## Conclusions

This study found no statistically significant difference between ChatGPT and DeepSeek AI in generating patient education materials for stroke imaging modalities, despite minor variations across topics. ChatGPT generally produced content at a slightly higher grade level, except for the DSA section where DeepSeek AI scored higher. In terms of readability, DeepSeek AI provided more accessible content for non-contrast CT and DSA, while ChatGPT performed marginally better for DW-MRI. These findings suggest that both tools deliver comparably structured and reliable educational materials, with differences influenced more by the specific imaging modality than by the AI model itself. Therefore, selection between the two may depend on user preference or context-specific readability needs rather than overall performance.
